# Polypus of Rectum

**Published:** 1889-05

**Authors:** Alfred Heath

**Affiliations:** 114 Ebury Street, London


### POLYPUS OF RECTUM.

MESSRS. EDITORS:—The following may be of some interest; it also shows how needless operations often are.

A gentleman, a few months since, came to me suffering from bleeding piles, for which I gave him Sulphur$^{200}$, with marked good results; all inconvenience of any kind disappeared.

At the beginning of November last he came complaining that the bowel protruded; on examination I found the bowel to be prolapsed so much as to be very inconvenient standing or riding.

I prescribed Merc. sol.$^{30}$, and advised a rectum pad to prevent its slipping down when exercising; in about a fortnight the pad could be entirely dispensed with, and was only worn on riding, as a precaution, but was needless; a friend of this gentleman advised his getting a *good* opinion.

He went to one of the first surgeons of London (I give the name, but not for publication) who, after examination, said that apart from the prolapsus, there was a polypus high up in rectum.

I thought it not unlikely that the tumor (for I found there was a small one) was the cause of the prolapsus, and I pointed out that if such were the case, the prolapsus being apparently cured, that the polypus must be better also, but nothing would satisfy him, and it was arranged the operation should take place immediately. Accordingly, one week after examination by this specialist, he came to operate, but, to his astonishment, *found there was no polypus to remove; he stated that it must have removed itself, and passed away, further, that the prolapsus also was gone;* this we knew before; accordingly not to lose his time, he removed three piles. Now as there had been no trouble from hæmorrhoids for some months, this was, probably, quite unnecessary, even from an allopathic point of view.

Yours truly,

ALFRED HEATH, F. L. S.

114 EBURY STREET, LONDON, January 19th, 1889.

